# Early Interdisciplinary Supportive Care in Patients With Previously Untreated Metastatic Esophagogastric Cancer: A Phase III Randomized Controlled Trial

**DOI:** 10.1200/JCO.20.01254

**Published:** 2021-01-08

**Authors:** Zhihao Lu, Yu Fang, Chang Liu, Xiaotian Zhang, Xiaowei Xin, Yi He, Yanshuo Cao, Xi Jiao, Tianqi Sun, Ying Pang, Yanli Wang, Jun Zhou, Changsong Qi, Jifang Gong, Xicheng Wang, Jian Li, Lili Tang, Lin Shen

**Affiliations:** ^1^ Department of Gastrointestinal Oncology, Key Laboratory of Carcinogenesis and Translational Research (Ministry of Education/Beijing), Peking University Cancer Hospital & Institute, Hai-Dian District, Beijing, China; ^2^ Department of Clinical Nutrition, Peking University Cancer Hospital & Institute, Hai-Dian District, Beijing, China; ^3^ Department of Psycho-Oncology, Peking University Cancer Hospital & Institute, Hai-Dian District, Beijing, China; ^4^ Precision Scientific (Beijing) Co, Ltd, Hai-Dian District, Beijing, China

## Abstract

Effective interventions to improve prognosis in metastatic esophagogastric cancer (EGC) are urgently needed. We assessed the effect of the early integration of interdisciplinary supportive care for patients with metastatic EGC on overall survival (OS).

**PATIENTS AND METHODS:**

An open-label, phase III, randomized, controlled trial was conducted at Peking University Cancer Hospital & Institute. Patients with previously untreated metastatic EGC were enrolled. Patients were randomly assigned (2:1) to either early interdisciplinary supportive care (ESC) integrated into standard oncologic care or standard care (SC). ESC was provided by a team of GI medical oncologists, oncology nurse specialists, dietitians, and psychologists; patients in the SC group received standard oncologic care alone. The primary end point was OS in the intention-to-treat population.

**RESULTS:**

Between April 16, 2015, and December 29, 2017, 328 patients were enrolled: 214 in the ESC group and 114 in the SC group. At the data cutoff date of January 26, 2019, 15 (5%) patients were lost to follow-up. The median number of cycles of first-line chemotherapy was five (interquartile range [IQR], 4-7) in the ESC group and four (IQR, 2-6) in the SC group. The median OS was 14.8 months (95% CI, 13.3 to 16.3) in the ESC group and 11.9 months (95% CI, 9.6 to 13.6) in the SC group (hazard ratio, 0.68; 95% CI, 0.51 to 0.9; *P* = .021).

**CONCLUSION:**

The early integration of interdisciplinary supportive care is an effective intervention with survival benefits for patients with metastatic EGC. Further optimization and standardization are warranted.

## INTRODUCTION

Esophagogastric cancer (EGC) is the second leading cause of cancer-related death worldwide.^1^ More than one million patients are diagnosed with EGC annually worldwide, and approximately 50% of cases occur in China.^1,2^ Patients with EGC are often diagnosed at an advanced stage and have a median overall survival (OS) of approximately 10 months.^3,4^ In the past decade, trastuzumab has been the only approved first-line agent for a subset of patients with human epidermal growth factor receptor 2 (HER2)-positive advanced esophagogastric adenocarcinoma,^3^ while other targeted therapies and immunotherapies are still under investigation. Therefore, there is an urgent need for effective interventions to improve the prognosis of metastatic EGC, especially in the first-line setting.

**CONTEXT**

**Key Objective**
To investigate the effect of early integration of nutritional and psychological intervention combined with standard oncologic care for patients with previously untreated metastatic esophagogastric cancer (EGC). To the best of our knowledge, this is the first study of early interdisciplinary supportive care (ESC) for metastatic EGC.
**Knowledge Generated**
ESC integrated with standard first-line chemotherapy demonstrated a survival benefit, with a hazard ratio of 0.68 in metastatic EGC.
**Relevance**
Our study provides new clinical evidence to support the early integration of nutritional and psychological supportive care into standard oncologic care for metastatic EGC.

Because of particulars of anatomy and complications from surgery or disease, EGC is characterized by a high incidence of malnutrition.^5,6^ Moreover, chemotherapy-related digestive side effects also compromise nutritional status.^7,8^ Cumulative evidence suggests that weight loss is a strong independent predictor of inferior survival in patients with various tumors.^9-11^ This association was even more notable in patients with EGC.^12^ In addition to impaired nutritional status, 24%-64% of patients with EGC suffer from psychological distress, which is also associated with malnutrition and even worse survival outcomes.^13-15^ These findings raise the question of whether supportive care in nutrition and psychology could alleviate symptoms, restore physical and psychological conditions, and eventually prolong OS in patients with metastatic EGC.

It is well established that early symptom monitoring and symptom management can improve quality of life (QoL)^16,17^ and even survival in patients with cancer.^18,19^ However, limited evidence has confirmed the survival benefit of nutritional or psychological supportive care in patients with cancer, especially in patients with metastatic EGC.^20-23^ Considering the higher prevalence of malnutrition and mood disorder in patients with EGC than in patients with other solid tumors, a feasible supportive care model adapted for metastatic EGC is urgently needed.^24,25^

Therefore, we prospectively conducted a randomized controlled trial to compare the clinical benefits of early integration of interdisciplinary supportive care, which included nutritional and psychological support, with standard anticancer treatment in patients with previously untreated metastatic EGC.

## PATIENTS AND METHODS

### Study Design and Participants

This study was an open-label phase III randomized controlled trial performed at Peking University Cancer Hospital & Institute.

Only inpatients were considered for inclusion. Eligible patients had histologically or cytologically confirmed esophageal squamous cell carcinoma (EC) or gastric or gastroesophageal junction adenocarcinoma (GC), were previously untreated, were 18 years or older, were diagnosed with metastatic disease within 8 weeks of study enrollment, had at least one measurable lesion per RECIST version 1.1, had a baseline Eastern Cooperative Oncology Group (ECOG) performance status of zero, one, or two, had an estimated life expectancy of more than 3 months, and had adequate cognitive and reading abilities.

This study was approved by the Ethics Committee of Peking University Cancer Hospital and was performed in accordance with the Declaration of Helsinki and Good Clinical Practice guidelines, as defined by the International Conference on Harmonization. No changes were made to the study design after the trial began. All patients provided written informed consent before study participation.

### Random Assignment and Masking

Patients were randomly assigned (2:1) to either the early interdisciplinary supportive care (ESC) combined with the standard oncologic care group (ESC group) or the standard oncologic care–alone group (standard care [SC] group). An interactive web response system was used for random assignment, and the patients were stratified according to the primary tumor site (EC or GC). Allocation was performed by an independent research nurse. This study was open-label, and neither the investigators nor the participants were masked to the intervention allocation.

### Procedures

**FIG A1.**
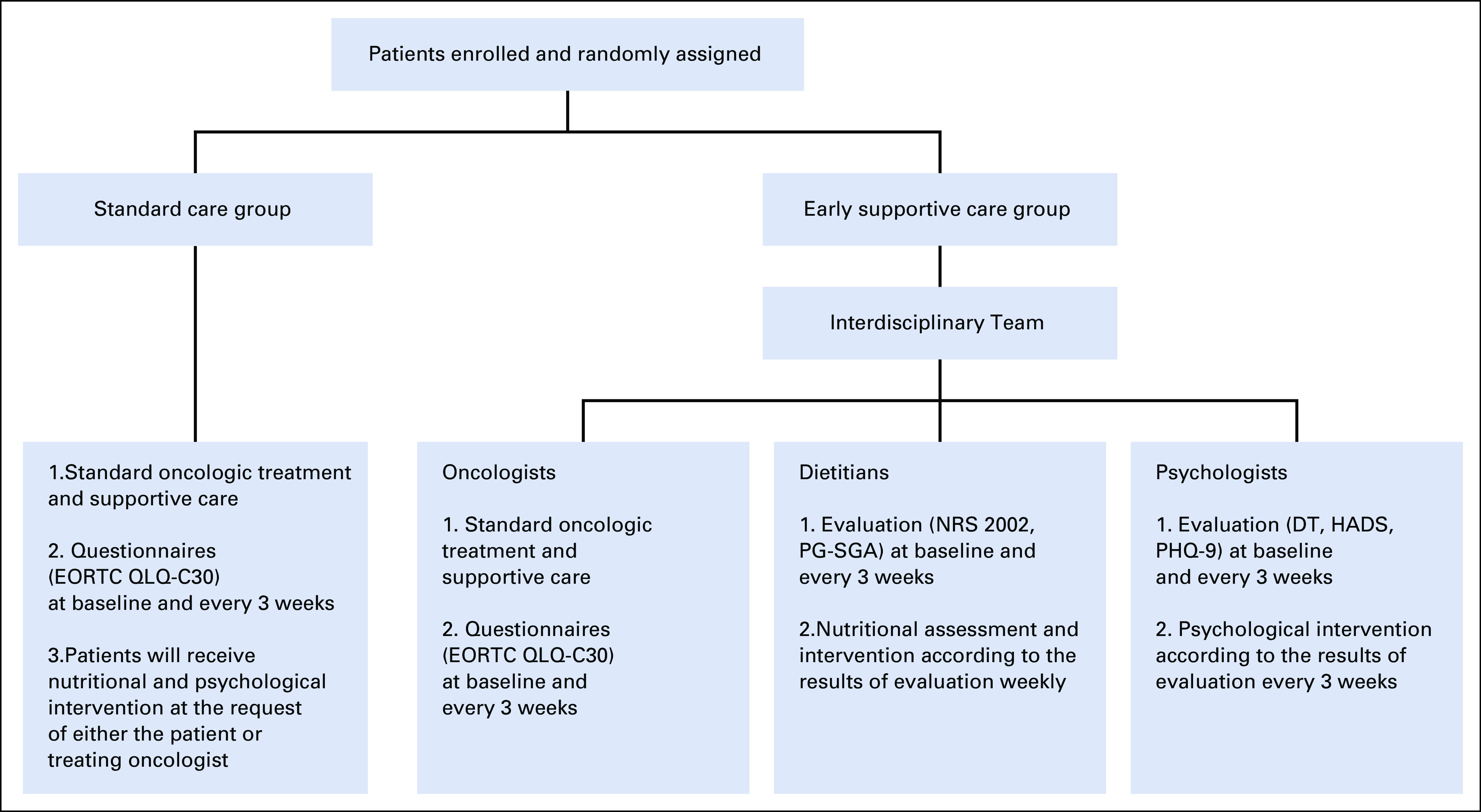
Early interdisciplinary supportive care team workflow. DT, distress thermometer; EORTC-QLQ-C30, European Organization for Research and Treatment of Cancer Quality-of-Life Questionnaire Core 30; NRS 2002, Nutritional Risk Screening 2002; PHQ-9, Patient Health Questionnaire-9; PG-SGA, Patient-Generated Subjective Global Assessment.

The workflow of the ESC team is shown in the Appendix Figure A1 (online only). Medical decisions were made by GI medical oncologists according to the *National Comprehensive Cancer Network Guidelines*^26^ and *Management of Gastric Cancer in Asia*^27^ combined with patient preference.

In the ESC group, based on the standard of care, the patients were taken by the research nurse to meet the interdisciplinary ESC team, which included a GI medical oncologist, an oncology specialty nurse, a dietitian, and a psychologist. The first meeting was arranged within 14 days prior to the initiation of chemotherapy, and subsequent meetings occurred every 3 weeks during the first-line treatment. The patients who were randomly assigned to the SC group were not scheduled for nutrition or psychology assessment unless either the patient or the treating oncologist requested an assessment. Patients in the SC group who received nutrition or psychology consultation did not cross over to the ESC group.

In the ESC group, the early supportive care intervention had two major components: nutrition and psychology. (1) After random assignment, nutritional risk screening (NRS) and nutritional assessments were completed by dietitians and included the NRS 2002 and Patient-Generated Subjective Global Assessment (PG-SGA), information on dietary intake, a physical examination, and a hematology test for each patient. Nutritional interventions were initiated according to the assessment results. (2) Psychological assessment was performed using a distress thermometer (DT), the Hospital Anxiety and Depression Scale (HADS), and the Patient Health Questionnaire-9 (PHQ-9). Psychologists conducted individual psychotherapy and family psychotherapy with each patient and his or her family members, and psychotropic interventions were provided when necessary. More details on the nutritional and psychological interventions are described in the Data Supplement (online only).

QoL was assessed in both groups every 3 weeks during first-line treatment, while nutrition and psychological assessment was conducted only for the patients in the ESC group. To ensure that every patient received coordinated interventions, members of the ESC team met weekly to discuss trial-related issues and potential solutions to improve the process. Additional study procedure details are listed in the Data Supplement.

### Outcomes

The primary end point was OS in the intention-to-treat (ITT) population, defined as the time from random assignment to death from any cause. Prespecified subgroup analyses assessed the association between OS and the stratification factors: EC and GC. Secondary end points included the change in QoL scores, based on the European Organization for Research and Treatment of Cancer Quality-of-Life Questionnaire Core 30 (EORTC QLQ C30) version 3.0, from baseline to 9 weeks, the objective response rate (ORR), and adverse events (AEs).

The EORTC QLQ-C30 is a 30-item questionnaire that consists of five function scales (physical, role, cognitive, emotional, and social), three symptom scales (fatigue, pain, and nausea and vomiting), six single items (dyspnea, insomnia, appetite loss, constipation, diarrhea, and financial difficulties), and a global QoL scale. For the global QoL scale and the function scales, higher scores denote improved function; for the symptom scales and the single items, higher scores denote worse symptoms.

Tumor assessments were performed at baseline and every 6 weeks after the initiation of chemotherapy. Responses were categorized as complete response (CR), partial response (PR), stable disease, or progressive disease according to RECIST version 1.1. An objective response was determined when patients had PR or CR as the best overall response. AEs were collected according to the National Cancer Institute Common Terminology Criteria for Adverse Events version 4.0. Progression-free survival (PFS) was calculated as the time from random assignment to radiological disease progression or death from any cause. More details regarding the NRS 2002, PG-SGA, DT, HADS, and PHQ-9 are provided in the Data Supplement.

### Statistical Analysis

This study was designed to have 80% power to detect an OS hazard ratio (HR) of 0.68 (an increase in the median OS from 9.0 months to 13.3 months) in favor of ESC, with a one-sided type I error rate of 0.025. Considering the random assignment (2:1) and an overall predicted dropout rate of 5%, the required number of patients was 330.

OS was analyzed in the ITT population. QoL, ORR, PFS, and AEs were analyzed in the per protocol population.

Statistical analyses were performed using R 3.6.1.^28^ Baseline characteristics were summarized using median and interquartile range (IQR) for continuous variables or number and percentage for categorical variables. Differences in baseline characteristics between study groups were assessed using a two-sample *t*-test for continuous variables and Fisher's exact test for categorical variables.

OS and PFS were estimated using the Kaplan-Meier method. Treatment effect differences in OS and PFS were assessed using the log-rank test, stratified by the randomization stratification factor. HRs and their associated 95% CIs were calculated using the Cox proportional hazard model adjusted for sex, age, ECOG status, and primary tumor site. For subgroup analyses of OS, HRs and corresponding 95% CIs were calculated using the unstratified Cox proportional hazard model. The corresponding 95% CIs of ORR were calculated using the Clopper-Pearson method. All scales and items of the EORTC QLQ C30 were converted to a 100-point scale. The effect of ESC on QoL outcomes was assessed by multivariate regression with adjustment for baseline QoL scores. Assessments for nutrition (NRS 2002 and PG-SGA) and psychology (DT, HADS, and PHQ-9) characteristics were conducted only for patients in the ESC group, and a paired *t*-test was used to determine whether there was a statistically significant change between the baseline and week 9 scores. Fisher's exact test was used to assess whether the proportion of patients who experienced weight loss was significantly different between the SC and ESC groups.

## RESULTS

**FIG 1.**
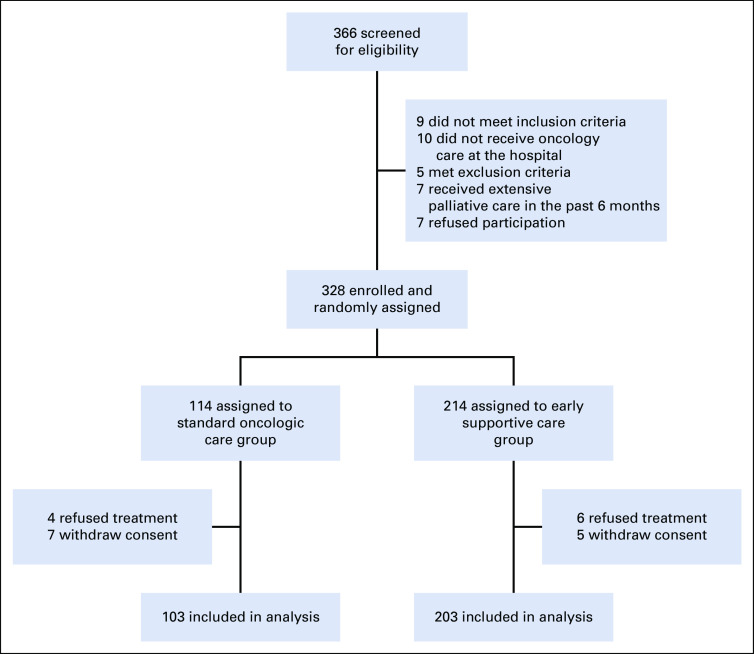
Trial profile.

Between April 16, 2015, and December 29, 2017, a total of 366 patients with metastatic esophageal or gastric cancer were screened for participation and 328 were enrolled and randomly assigned to receive ESC (n = 214) or standard oncologic care (n = 114; Fig 1). Finally, 328 patients were analyzed for OS, and 306 patients (203 patients in the ESC group and 103 in the SC group) were analyzed for secondary end points. Demographic and baseline clinical characteristics were well balanced between the study groups (Table 1). In the SC group, eight patients (7%) received a nutrition consultation, and 17 patients (15%) received a psychology consultation during the first 9 weeks.

**TABLE 1.**
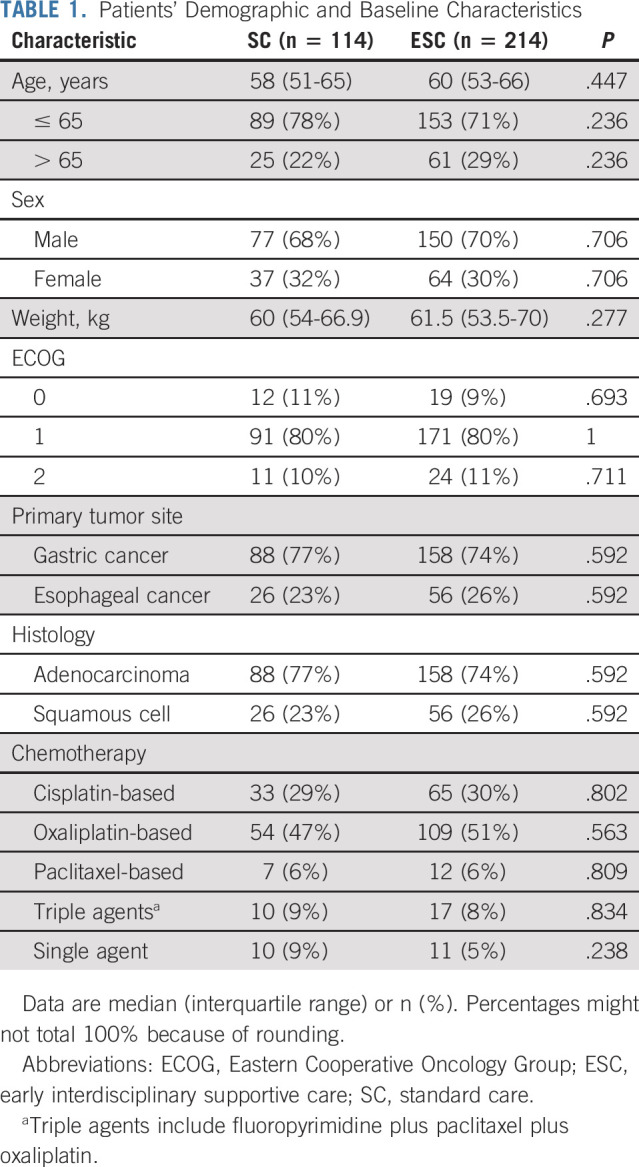
Patients’ Demographic and Baseline Characteristics

**FIG 2.**
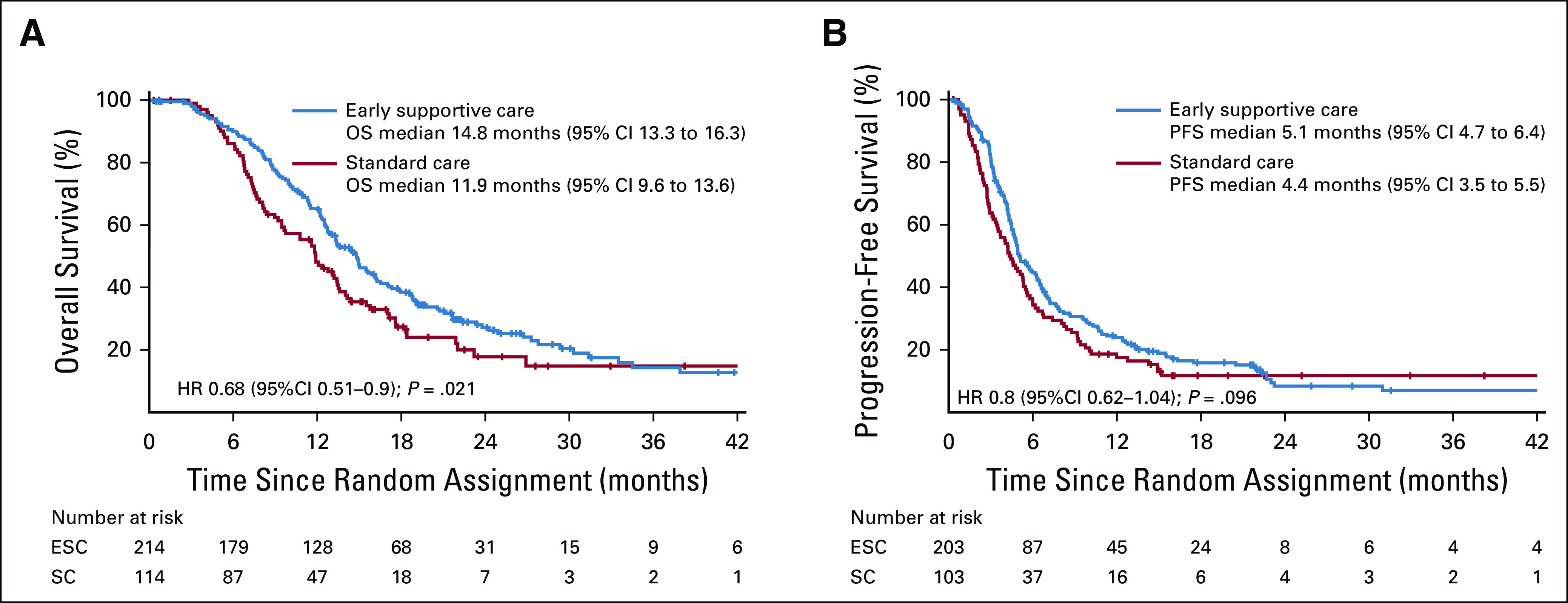
Kaplan-Meier analyses of survival. (A) OS. (B) PFS. ESC, early interdisciplinary supportive care; HR, hazard ratio; OS, overall survival; PFS, progression-free survival; SC, standard care.

**FIG 3.**
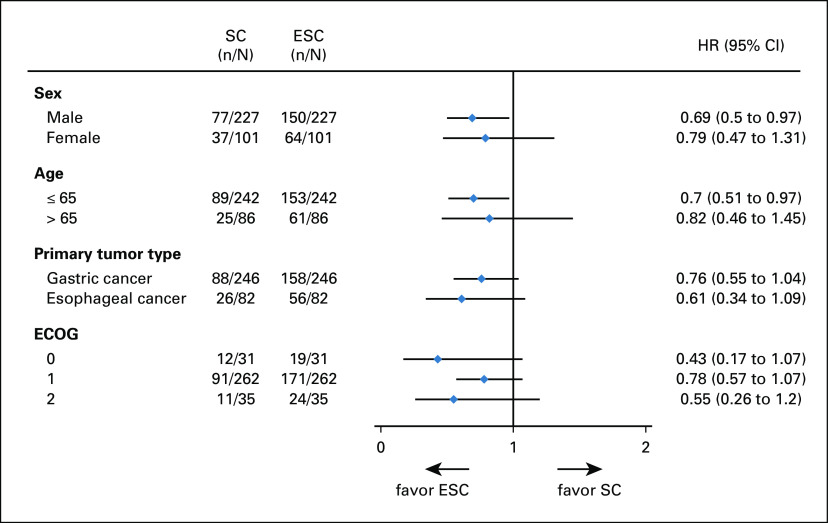
HRs and 95% CIs for overall survival by subgroup. The forest plot shows the HRs and the corresponding 95% CIs for ESC compared with SC. HRs were calculated using the unstratified Cox proportional hazard model. ECOG, Eastern Cooperative Oncology Group; ESC, early interdisciplinary supportive care; HR, hazard ratio; SC, standard care.

At the cutoff date (January 26, 2019), 81 (25%) patients were still alive and 15 (5%) patients were lost to follow-up. The median number of cycles of first-line chemotherapy received was five (IQR, 4-7) in the ESC group and four (IQR, 2-6) in the SC group. The median OS was 14.8 months (95% CI, 13.3 to 16.3) for patients assigned to the ESC group compared with 11.9 months (95% CI, 9.6 to 13.6) for patients in the SC group (HR, 0.68; 95% CI, 0.51 to 0.9; *P* = .021; Fig 2). A preplanned exploratory analysis according to the primary tumor site showed that OS was longer in the ESC group than in the SC group in both the GC (HR, 0.76; 95% CI, 0.55 to 1.04) and EC (HR, 0.61; 95% CI, 0.34 to 1.09) subpopulations (Fig 3).

The median PFS was 5.1 months (95% CI, 4.7 to 6.4) for patients who received ESC compared with 4.4 months (95% CI, 3.5 to 5.5) for patients who received SC (HR, 0.8; 95% CI, 0.62 to 1.04; *P* = .096; Fig 2). The ORRs of the ESC group and the SC group were 32% (95% CI, 25.7 to 38.9) and 27% (95% CI, 18.9 to 36.8), respectively (Appendix Table A1, online only). AEs were reported in 181 of 203 (89%) patients who received ESC and 89 of 103 (86%) who received SC. There were no significant differences in the frequency of AEs between the two groups (Appendix Table A2, online only). No intervention-related AEs or unintended effects occurred in either group.

**FIG 4.**
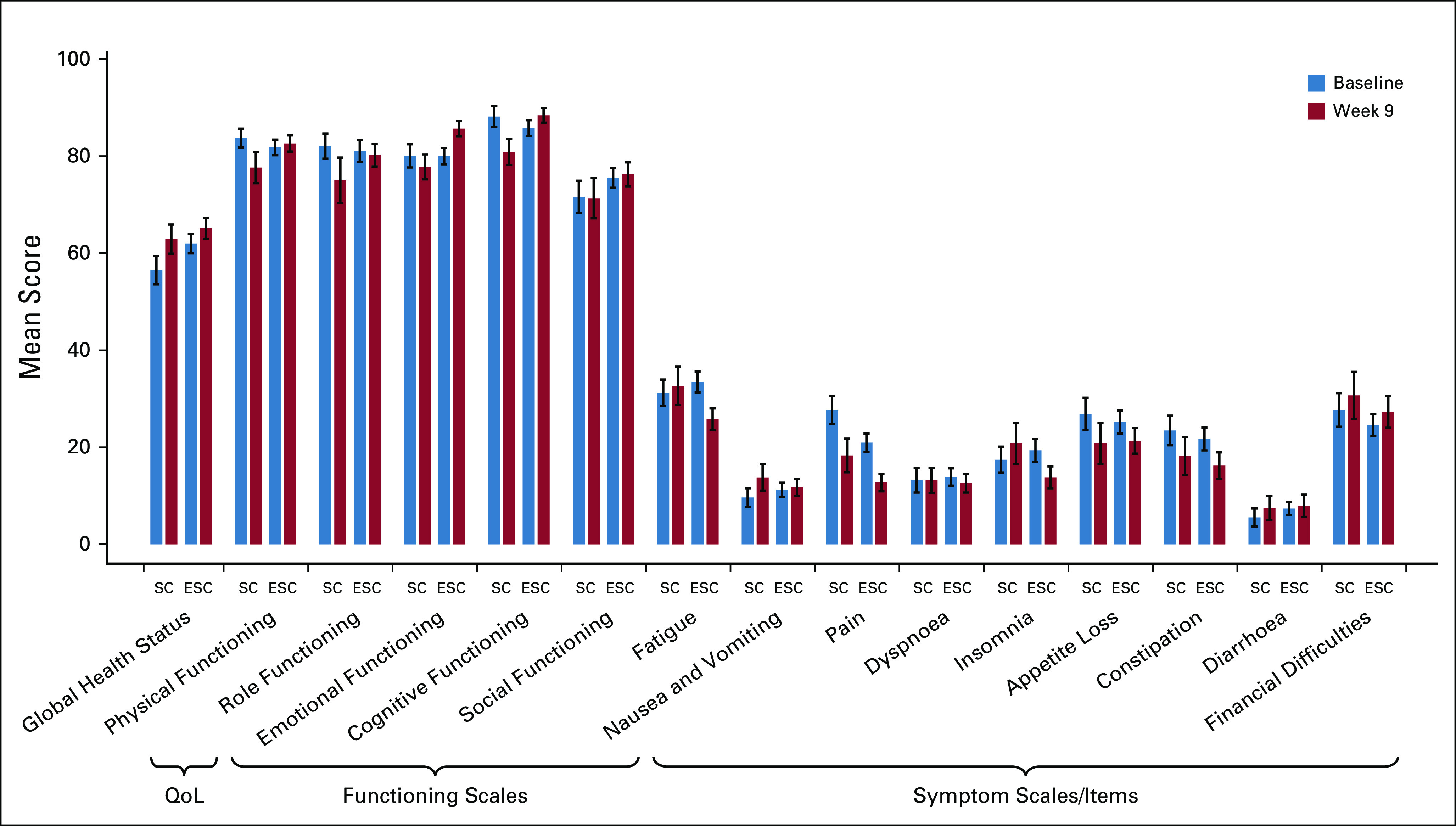
Mean quality-of-life scores at baseline and week 9 in the SC group and the ESC group. QoL (EORTC QLQ-C30) was analyzed in the per protocol population. Bars represent the mean scores on the EORTC QLQ-C30 at baseline (blue) and week 9 (red) for both the SC group and the early supportive care group. The error bar represents SE. For the global health status and functioning scales, higher scores denoted improved function; for symptom scales and single items, higher scores denoted worse symptoms. The effect of ESC on QoL outcomes was assessed by multivariate regression with adjustment for baseline QoL scores. The adjusted effect of ESC was significant for emotional functioning (5.87; 95% CI, 0.05 to 11.69; *P* = .048) and cognitive functioning (5.77; 95% CI, 0.28 to 11.25; *P* = .039). EORTC QLQ-C30, European Organization for Research and Treatment of Cancer Quality-of-Life Questionnaire Core 30; ESC, early interdisciplinary supportive care; QoL, quality of life; SC, standard care.

The results for overall QoL, functioning, and symptoms at baseline and 9 weeks are presented in Figure 4. The compliance at 9 weeks was 55% in the ESC group versus 51% in the SC group. With the adjustment for baseline QoL scores by multivariate regression, the ESC interventions had a significant effect on emotional functioning and cognitive functioning at week 9. The adjusted effects of ESC on emotional functioning and cognitive functioning were estimated to be 5.87 (95% CI, 0.05 to 11.69; *P* = .048) and 5.77 (95% CI, 0.28 to 11.25; *P* = .039), respectively.

**FIG A2.**
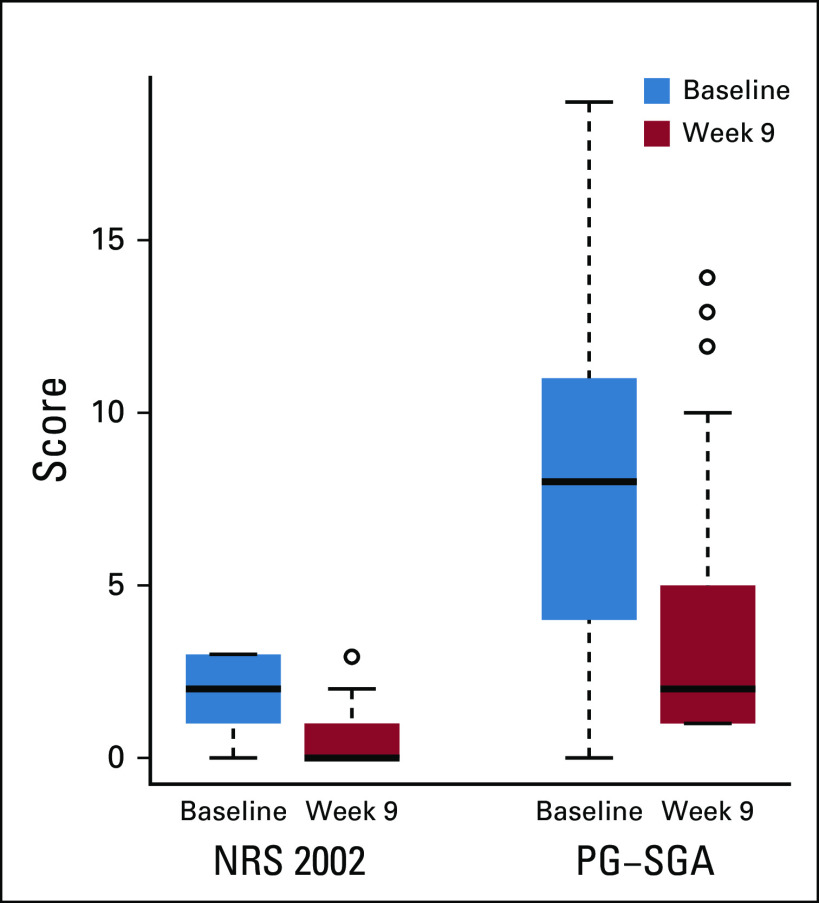
Boxplots for NRS 2002 and PG-SGA scale scores at baseline and week 9 in the ESC group. NRS 2002 and PG-SGA scales were analyzed at baseline (blue) and week 9 (red) in the early supportive care group. Boxplots for the NRS2002 and PG-SGA scales display the total range, interquartile range (box) and median (line). Significant improvements were observed in the mean score from baseline to week 9 in NRS 2002 (1.61, 95% CI, 1.45 to 1.78, *v* 0.73, 95% CI, 0.54 to 0.91, *P* < .001) and PG-SGA (7.55, 95% CI, 6.95 to 8.15, *v* 3.40, 95% CI, 2.90 to 3.90, *P* < .001). NRS 2002, Nutritional Risk Screening 2002; PG-SGA, Patient-Generated Subjective Global Assessment.

**FIG A3.**
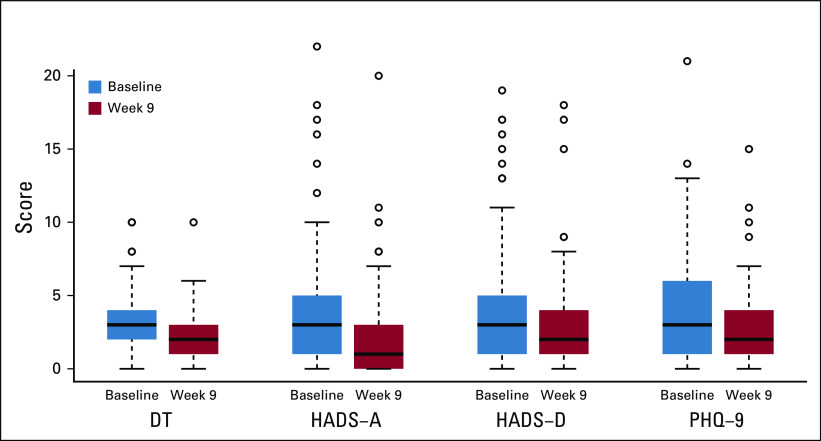
Boxplots for DT, HADS-Anxiety, HADS-Depression, and PHQ-9 scales at baseline and week 9 in the ESC group. DT, HADS-A, HADS-D and PHQ-9 scales were analyzed at baseline (blue) and week 9 (red) in the early supportive care group. Boxplots for the DT, HADS-A, HADS-D and PHQ-9 scales display the total range, interquartile range (box) and median (line). Significant improvements were observed in the mean score from baseline to week 9 in DT (3.22, 95% CI, 2.91 to 3.52, *v* 2.28, 95% CI, 1.98 to 2.58, *P* < .001), HADS-Anxiety (3.54, 95% CI, 3.01 to 4.08, *v* 2.26, 95% CI, 1.77 to 2.75, *P* < .001), HADS-Depression (3.92, 95% CI, 3.35 to 4.49, *v* 2.67, 95% CI, 2.13 to 3.20, *P* < .001), and PHQ-9 (4.05, 95% CI, 3.51 to 4.59, *v* 2.73, 95% CI, 2.24 to 3.22, *P* < .001). DT, Distress Thermometer; HADS-A, Hospital Anxiety and Depression Scale-anxiety; HADS-D, Hospital Anxiety and Depression Scale-depression; PHQ-9, Patient Health Questionnaire-9.

**FIG A4.**
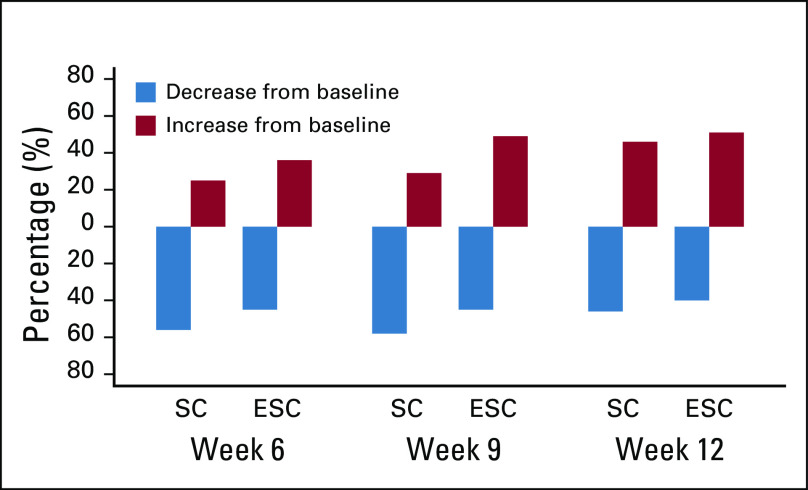
Proportion of weight change in patients from baseline to week 6, week 9, and week 12 in the SC group and the ESC group. Bars represent the percentage of patients with decreased (blue) or increased (red) weight compared with baseline. ESC, early supportive care; SC, standard care.

At baseline, 196 (97%) patients in the ESC group received NRS with the NRS 2002 and assessment with the PG-SGA scale, and 132 of 196 (67%) patients were re-evaluated at week 9. The results showed significant improvements in the mean NRS 2002 and PG-SGA scores from baseline to week 9 (Appendix Fig A2, online only; Appendix Table A3, online only). In the ESC group, 178 (88%) patients underwent psychological assessments with the DT, HADS-Anxiety (HADS-A), HADS-Depression (HADS-D), and PHQ-9 scales at baseline. A total of 129 of 178 (72%) patients underwent reassessment at week 9, and we observed a significant decrease in mean scores from baseline to week 9 on the DT, HADS-A, HADS-D, and PHQ-9 scales (Appendix Fig A3, online only; Appendix Table A3). In addition, 58% (36 of 62) of patients in the SC group presented weight loss at week 9, while 45% (57 of 126) of patients in the ESC group presented weight loss (Appendix Fig A4, online only). The proportion of patients who experienced weight loss was significantly different between the SC and ESC groups at week 9 (*P* = .032).

## DISCUSSION

This randomized phase III trial showed that the integration of ESC with standard oncologic care significantly prolonged OS, with an HR of 0.68 compared with standard oncologic care alone, in patients with metastatic EGC. For the secondary end points, despite similar responses to systemic therapy and safety profiles between the two groups, alleviation of emotional function and cognitive function at 9 weeks, and improvements in nutritional and psychological status were observed in the interdisciplinary ESC group. Importantly, compared with current first-line anticancer agents,^3,29^ ESC intervention integrated with standard chemotherapy showed a promising survival benefit in patients with metastatic EGC. Therefore, this prospective randomized study provides clinical evidence that the integration of ESCs into standard oncologic care for metastatic EGC may optimize the effect of anticancer therapy.

It is well known that patients with EGC have the highest presence of malnutrition,^30^ and nutritional problems are always associated with psychological stress in patients with cancer.^15^ These two factors play an important role in patients' tolerance of anticancer treatments and in enhancing their confidence when struggling with long-term illness. More importantly, nutritional and psychological status is closely correlated with immune function,^31,32^ which can compromise the treatment effect of targeted therapies or immunotherapies in patients with metastatic EGC. In first-line treatments, trastuzumab was the only targeted agent that has shown promising results, with reducing the risk of death by 26%, which accounts for only 22.1% of patients with metastatic EGC.^3^ Other targeted therapies and immunotherapies are still under investigation. Therefore, beyond the development of novel anticancer agents, it is urgent to explore other interventions to improve survival in patients with metastatic EGC. Previous studies have demonstrated the benefits of supportive care in terms of QoL, symptom control, and mood management.^16,18^ In 2017, Basch et al^19^ identified the survival benefits of supportive care based on patient-reported outcomes, which highlighted the importance of early symptom control for preventing worse consequences in patients with cancer. To the best of our knowledge, the present study is the first to demonstrate the survival benefits of early interdisciplinary nutritional and psychological supportive care when combined with standard oncologic care in patients with metastatic EGC.

A potential mechanism of prolonged OS is the improvement of nutritional and psychological status in the ESC group. Depression and anxiety have been found to be associated with a significantly increased risk of cancer-specific mortality.^33^ Meanwhile, individualized nutritional intervention was demonstrated to improve 30-day mortality.^34^ These impacts may be due to the influence of nutritional and psychological status on the immune response,^31,32^ which is important for improvement in the long-term prognosis. However, few studies have specifically investigated the use of both supports in patients with cancer. In our study, we enrolled only patients with EGC, provided both nutritional and psychological interventions, and, finally, observed the survival benefit, which further confirmed the effect of nutritional and psychological support for patients with EGC.

Given the progressive nature of the disease, improving QoL is a formidable challenge for patients with EGC.^35^ Although the global health status did not show a difference between the ESC group and SC group at week 9, emotional function and cognitive function were significantly improved in the ESC group. A previous meta-analysis also showed that nutritional interventions for ESC had a small effect on QoL,^36,37^ which was concordant with our results. Meanwhile, Temel et al^38^ reported heterogeneity in QoL changes between study groups in patients with lung cancer and GI cancer, which also supports our findings.

Our study has several strengths. First, we employed survival outcomes as end points for the first time in an ESC-related trial in patients with metastatic EGC and demonstrated a remarkable survival benefit from nutritional and psychological intervention integrated with standard chemotherapy, which provides direct evidence to improve current clinical practices for those patients. Second, compared with current anticancer treatments, the ESC intervention showed a promising survival benefit, which demonstrates the central role of such interventions in metastatic EGC treatment. Third, we established a simple ESC model for metastatic EGC that may promote the development of ESC programs for these patients.

Several limitations exist. First, this trial was conducted in a single institution that included only Chinese patients, which may limit the generalizability of our results to different races and other settings. Further optimization of this supportive care model is needed to adapt to different local resources and traditions. Second, there was potential bias because participants and investigators were not masked to group assignment. Third, the dropout rate by week 9 was 33% for nutritional interventions and 28% for psychological interventions. Excessive negative perceptions and stigmatization of cancers have existed for a long time among Chinese patients with cancer and their families, which may be the major cause of poor compliance. Finally, based on the impact of nutritional and psychological disorders on the immune response, further exploration of supportive care and immune biomarkers is needed.

In conclusion, we established a simple model of ESC that showed improved survival in patients with metastatic EGC. Our study provides new clinical evidence supporting the early integration of nutritional and psychological supportive care into standard oncologic care for those with metastatic EGC. Further optimization and standardization are warranted.

## Figures and Tables

**TABLE A1.**
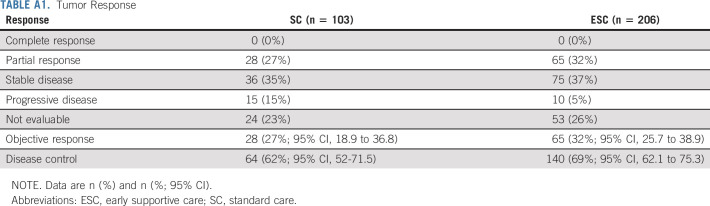
Tumor Response

**TABLE A2.**
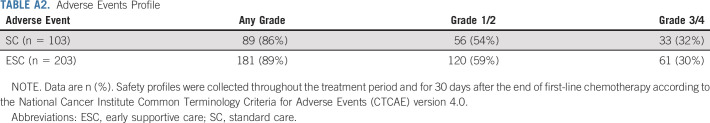
Adverse Events Profile

**TABLE A3.**
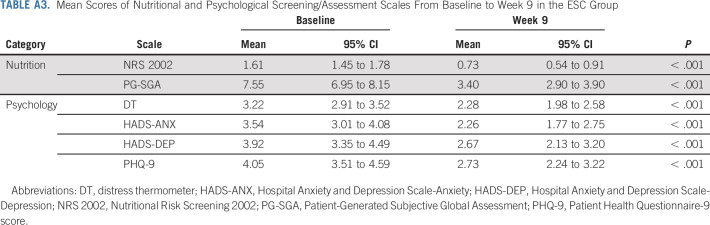
Mean Scores of Nutritional and Psychological Screening/Assessment Scales From Baseline to Week 9 in the ESC Group
